# The Quality of Marriage Index (QMI): a validation study in infertile patients

**DOI:** 10.1186/s13104-019-4438-2

**Published:** 2019-08-14

**Authors:** Saman Maroufizadeh, Amir Almasi-Hashiani, Payam Amini, Mahdi Sepidarkish, Reza Omani-Samani

**Affiliations:** 1 School of Nursing and Midwifery, Guilan University of Medical Sciences, Rasht, Iran; 2 Department of Epidemiology, School of Health, Arak University of Medical Sciences, Arak, Iran; 3 Department of Biostatistics and Epidemiology, School of Public Health, Ahvaz Jundishapur University of Medical Sciences, Ahvaz, Iran; 4 Department of Biostatistics and Epidemiology, Babol University of Medical Sciences, Babol, Iran; 5 Department of Medical Ethics and Law, Reproductive Biomedicine Research Center, Royan Institute for Reproductive Biomedicine, ACECR, Tehran, Iran

**Keywords:** Quality of Marriage Index, Marital satisfaction, Reliability, Validity, Infertility

## Abstract

**Objective:**

Infertility can have a considerable effect on a person’s marital satisfaction. The Quality of Marriage Index (QMI) is a self-report inventory to measure global perceptions of marital satisfaction. The current study examined the reliability and validity of the Persian language version of QMI in a sample of infertile patients.

**Results:**

The mean QMI total score was 36.54 ± 6.87. The internal consistency of the scale was good, with a Cronbach’s alpha of 0.922. All inter-item correlations and item-total correlations were also in acceptable range. The confirmatory factor analysis results provided evidence for unidimensionality of the scale (χ^2^/df = 3.10; GFI = 0.97; CFI = 0.99; NFI = 0.99; RMSEA = 0.091 and SRMR = 0.020). The convergent validity of the QMI was demonstrated via significant correlations with measures of the Relationship Assessment Scale, Kansas Marital Satisfaction Scale, and Couples Satisfaction Index-4 Item. These correlations also tended to be larger than correlations with measures of Hospital Anxiety and Depression Scale and Perceived Stress Scale-4 Item. Among demographic/fertility variables, only infertility duration was negatively correlated to QMI scores. In sum, the QMI is a reliable and valid brief inventory for measuring overall marital satisfaction in infertile patients.

*Trial registration* This was a cross-sectional study (NOT clinical trial); thus, the trail registration number is not required for the present study

## Introduction

Relationship satisfaction is the amount of a person’s feeling about his/her intimate relationship [1]. As a part of relationship satisfaction assessment, the quality of marriage has been introduced as a general evaluation of marriage in which factors such as various features of marriage, attitudes, behaviors and communication patterns are utilized [2, 3]. Some relationship characteristics such as the amount of satisfaction with the relationship, the type of attitudes towards the partner, and low levels of aggression and hostile can be used to investigate the quality of marriage [3, 4]. It has been shown that quality of marriage is associated with health problems and well-being, the feeling of happiness, economic factors, psychological complications, and general aspects of quality of life [4–7]. However, the assessment of marital quality among infertile patients is along with several complications. Infertility, which is defined as a failure to conceive after 12 months of unprotected intercourse, is followed by numerous psychological and mental health problems including depression, stress, anxiety, sexual dysfunction, and poor marital satisfaction, well-being, and quality of life [8–13].

A number of self-report tools have been introduced and used to evaluate the marital quality such as Marital Adjustment Test (MAT), Kansas Marital Satisfaction Scale (KMSS), Dyadic Adjustment Scale (DAS), Couples Satisfaction Index (CSI), Relationship Assessment Scale (RAS), Quality Marriage Index (QMI) [14]. The QMI which was developed by Norton [15], is a six-item measure of marital satisfaction. This instrument has been used among general populations [16, 17], cardiovascular [18], cancer [19], fertile couples [20], military veterans [21], and many of other samples. This scale is appropriate to check how agreement contributes in the relationship and similarity of attitudes within the couples [15]. Moreover, the brevity of the instrument in comparison to other tools can be a considerable advantage so that large populations can be assessed in a short period of time. Although, there is a controversy in reporting the amount of the QMI reliability, a meta-analysis study exposed an average strong reliability of 0.94 across several studies [22]. Moreover, QMI scores among women are more reliable than in men [22]. It has been argued that although the QMI has strong intrinsic psychometric properties and performs better for longer term relationships [22].

Regarding the psychological problems among infertile couples and the necessity of their marital quality assessment, this study aims to examine the reliability and validity of the QMI among a sample of infertile patients.

## Main text

### Methods

#### Participants and study design

In this cross-sectional study, infertile patients referring to infertility treatment center of Royan Institute, Tehran, Iran were invited to take part in the study. The data were collected via convenience sampling method from February to May 2017. Patients had to meet the following criteria to be eligible for the study: (1) experiencing infertility problems; (2) in a heterosexual marriage; (3) 18 years or older; (3) willingness to participate in the research; (4) ability to read, and write in Persian. In total, 254 infertile patients agreed to take part and filled out the questionnaires completely.

#### Measures

##### Quality of Marriage Index (QMI)

The QMI is a brief self-report instrument that measures marital quality [15]. The scale consists of 6 positively worded items that are rated on a 10-point Likert scale, ranging from 1 to 10 for the last item, and on 7-point Likert scale, ranging from 1 to 7 for the other five items. Total scores range from 6 to 45, with higher scores reflecting better marital quality.

##### Relationship Assessment Scale (RAS)

The RAS is a brief, 7-item self-report instrument that measures relationship satisfaction [1]. Each item is rated on a 5-point Likert scale ranging from 1 to 5. Total scores range from 7 to 35, with higher scores reflecting better relationship satisfaction. The Persian language version of RAS has been validated among infertile patients [23]. In this study, the Cronbach’s alpha coefficient of the RAS was 0.828.

##### Kansas Marital Satisfaction Scale (KMSS)

The KMMS is a brief, 3-item self-report instrument that measures marital satisfaction [24]. Each item is rated on a 7-point Likert scale ranging from 1 (extremely dissatisfied) to 7 (extremely satisfied). Total scores range from 3 to 21, with higher scores reflecting greater marital satisfaction. The Persian language version of KMSS has been validated among infertile patients [25]. In this study, the Cronbach’s alpha coefficient of the KMSS was 0.901.

##### The 4-Item Couples Satisfaction Index (CSI-4)

The CSI-4 is a widely used self-report instrument derived from the original 32 item CSI (CSI-32) that measures relationship satisfaction [16]. The scale consists of four positively worded items that are rated on a 7-point Likert scale, ranging from 0 (extremely unhappy) to 6 (perfect) for the first item, and on 6-point Likert scale, ranging from 0 (not at all true) to 5 (all of the time) for the other three items. Total scores range from 0 to 21, with higher scores reflecting better relationship satisfaction. In this study, the Cronbach’s alpha coefficient of the CSI-4 was 0.846.

##### Hospital Anxiety and Depression Scale (HADS)

The HADS is a commonly used self-report instrument consisting 14 items designed to measure both anxiety (HADS-A, 7 items) and depression (HADS-D, 7 items) [26]. Each item is rated on a 4-point Likert scale ranging from 0 to 3. Both subscale scores range from 0 to 21, with higher scores reflecting greater anxiety and depression. The Persian language version of HADS has been validated among infertile patients and widely used in this population [8, 9]. In this study, the Cronbach’s alpha coefficient of the HADS-A and HADS-D were 0.842 and 0.721, respectively.

##### Perceived Stress Scale-4 Item (PSS-4)

The PSS-4 is a widely used self-report instrument derived from the original 14 item PSS (PSS-14) that measures “the degree to which situations in one’s life over the last month are appraised as unpredictable, uncontrollable and overloading” [27]. Each item is rated on a 5-point Likert scale, ranging from 0 (never) to 4 (very often). Total scores range from 0 to 16, with higher scores reflecting greater stress [27]. The Persian language version of PSS has been validated among infertile patients and adults with asthma [28, 29]. In this study, the Cronbach’s alpha coefficient of the PSS-4 was 0.555.

#### Statistical analysis

The confirmatory factor analysis (CFA), with maximum likelihood estimation method, was performed in order to evaluate the unidimensionality of QMI. The fit of the model was assessed using several goodness-of-fit indices including the Chi square/degree of freedom (χ^2^/df), the goodness of fit index (GFI), the compara-tive fit index (CFI), the normed fit index (NFI), the root mean square error of approximation (RMSEA), and the standardized root mean square residual (SRMR). Values of χ^2^/df < 5, GFI, CFI, and NFI > 0.90, and RMSEA and SRMR < 0.08 indicate acceptable fit to the data [30–33]. Cronbach’s alpha, inter-item correlation, and corrected-item total correlation were used to examine the internal consistency of the scale. To examine the convergent validity of the QMI, we calculated Pearson correlations between the QMI scores and the measures of the KMSS, RAS, CSI-4, HADS, and PSS-4. In addition, Pearson correlation coefficient, independent t test and one-way ANOVA were used to examine the relationship between QMI scores and demographic/fertility variables.

All statistical analyses were done with SPSS for windows, version 16.0 (SPSS Inc., Chicago, IL, USA) and LISREL 8.80 (Scientific Software International, Inc., Lincolnwood, IL, USA).

### Results

#### Participant characteristics

Patients had mean age of 32.09 years (SD = 6.55) and mean infertility duration of 4.85 years (SD = 3.73). Of the patients, 55.5% were females, 36.2% were university-educated, and 50.4% underwent first ART treatment. Infertility was due to a male or female factor in 35.8 and 21.7% of patients, respectively. In 19.3%, both male and female factors were observed, and 23.2% of the patients had unexplained infertility.

#### Descriptive statistics and internal consistency of the QMI

Item wording, descriptive statistics, and reliability analysis of the QMI are given in Table 1. The mean QMI total score was 36.54 ± 6.87 (range 14–45). The internal consistency of the QMI was good, with Cronbach’s alpha of 0.922. As seen in Table 2, Cronbach’s alpha did not significantly increase as a consequence of an item deletion. The corrected item-total correlations and the inter-item correlations ranged 0.766–0.858, and 0.618–0.796, respectively, which were in acceptable range.

**Table 1:** Items wording and descriptive statistics, and internal consistency of the QMI

		Mean	SD	Corrected item total correlation	Alpha if item deleted	Cronbach’s alpha
1	We have a good marriage	5.67	1.13	0.815	0.906	
2	My relationship with my partner is very stable	5.57	1.17	0.782	0.909	
3	Our marriage is strong	5.67	1.24	0.858	0.899	
4	My relationship with my partner makes me happy	5.92	1.19	0.766	0.911	
5	I really feel like part of a team with my partner	5.67	1.33	0.810	0.904	
6	The degree of happiness, everything considered, in our marriage is	8.04	1.89	0.772	0.924	
	QMI total score	36.54	6.87			0.922

*SD* standard deviation

**Table 2:** Relationship of QMI scores with demographic/fertility characteristics in infertile patients

	Mean ± SD or r	P
Age (years)	− 0.119	0.057
Duration of infertility (years)	− 0.158	0.012
Sex		0.607
Male	36.79 ± 7.23	
Female	36.34 ± 6.60	
Educational level		0.378
Primary	35.48 ± 7.43	
Secondary	36.96 ± 7.20	
University	36.78 ± 6.08	
Cause of infertility		0.239
Male factor	35.45 ± 7.22	
Female factor	36.87 ± 6.98	
Both	37.82 ± 6.78	
Unexplained	36.85 ± 6.19	
Failure of previous treatment		0.068
No (First treatment)	37.32 ± 6.65	
Yes	35.75 ± 7.03	
History of abortion		0.904
No	36.51 ± 6.89	
Yes	36.63 ± 6.87	

*SD* standard deviation; *r* correlation coefficients

#### Convergent Validity

As expected, there were strong correlations between QMI and measures of KMSS (r = 0.696), RAS (r = 0.700), and CSI-4 (r = 0.754). The QMI scores were also correlated with measures of HADS-A (r = − 0.242), HADS-D (r = − 0.406), and PSS-4 (r = − 0.376). According to these correlation coefficients, the correlations of QMI with measures of marital satisfaction (i.e., KMSS, RAS and CSI-4) were higher than the correlations with measures of anxiety, depression, and stress (i.e., HADS-A, HADS-D and PSS-4).

#### Confirmatory factor analysis

**Fig. 1:**
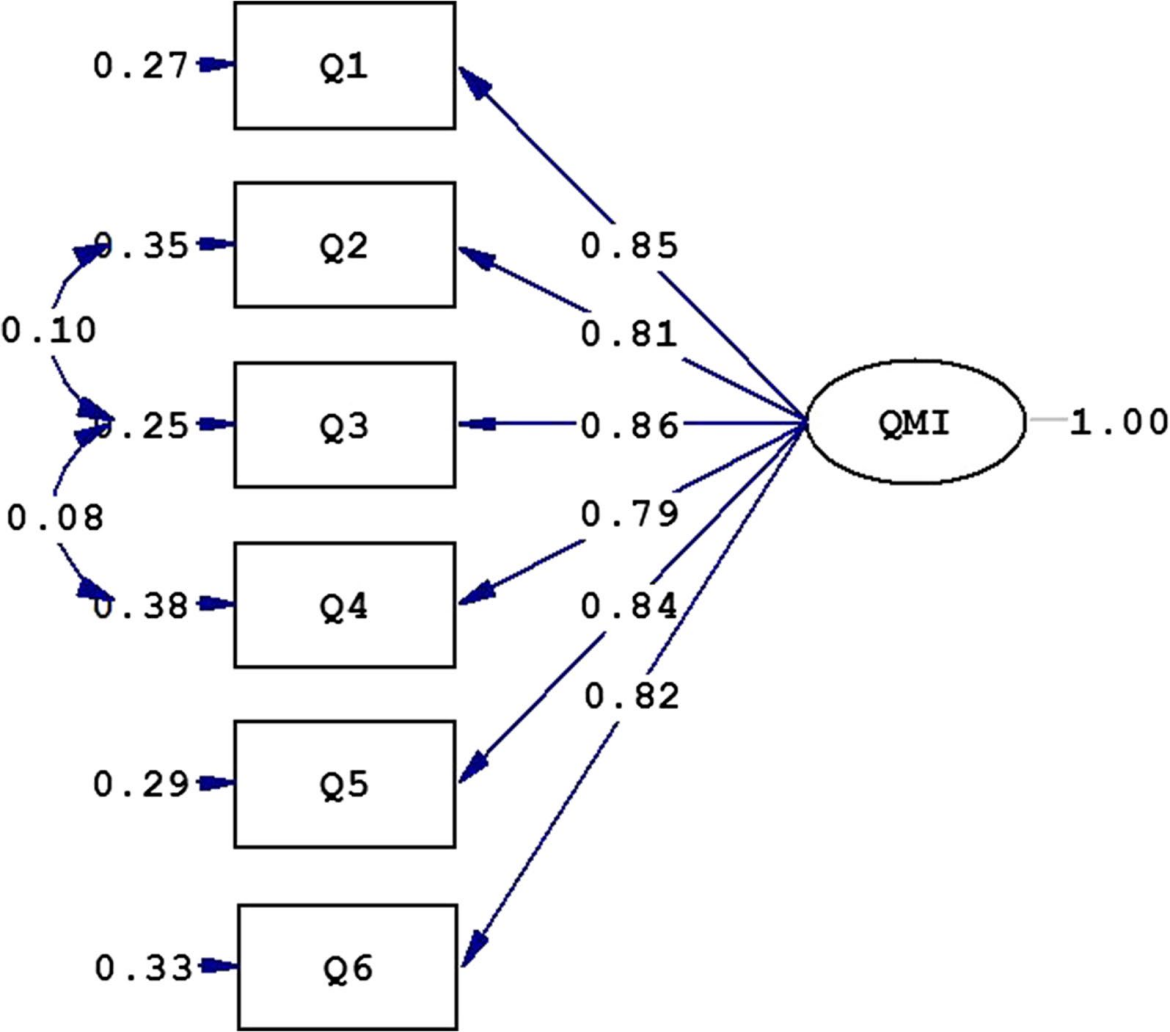
Unidimensional structure of the QMI in a sample of infertile patients

To test the unidimensionality of the QMI, the CFA was carried out. According to the goodness of fit indices, the fitness of the model was not good (χ^2^/df = 5.15; GFI = 0.94; CFI = 0.98; NFI = 0.97; RMSEA = 0.128 and SRMR = 0.028). Examination of the modification indices recommended allowing covariance between Item 2 and Item 3 as well as between Item 3 and Item 4 (Fig. 1). A better fit was obtained after allowing for these covariances (χ^2^/df = 3.10; GFI = 0.97; CFI = 0.99; NFI = 0.99; RMSEA = 0.091 and SRMR = 0.020). All factor loadings were significant and large (> 0.7, see Fig. 1).

#### Relationship of the QMI scores with demographic characteristics

As presented in Table 2, significant but low negative correlation was obtained between QMI scores and infertility duration (r = − 0.158, P = 0.012). Patients who had failure in previous treatment obtained lower QMI scores compared to patients undergoing first treatment, but this difference was not statistically significant (P = 0.068). Age, Sex, level of education, cause of infertility, and history of abortion were not related to QMI scores.

### Discussion

This study examined the psychometric characteristics of the QMI in a sample of infertile patients in Iran. The QMI demonstrated excellent internal consistency (α = 0.922), and alpha value did not increase when an item was deleted. All inter-item correlations and corrected item-total correlations were also within acceptable range, indicating good internal consistency. These findings are in line with what was reported in previous studies [22, 34]. The unidimensional structure of the QMI that we found in this study is consistent with Norton [15] theoretical conceptualization of the QMI. In a study conducted by Nazarinia and Schumm [35] among expectant and new Canadian mothers, factor analysis showed moderate support for unidimensional structure for a slightly modified version of QMI. Unfortunately, the literature in which this scale has been psychometrically studied is limited.

Evidence of convergent validity of the QMI was demonstrated by a pattern of correlations with the relevant measures of marital satisfaction and measures of anxiety, depression and stress that was in line with theoretical predictions. These results are in line with the previous studies which reported that the QMI scores were considerably related to measures of psychological distress and other instruments for assessing marital satisfaction and quality [16, 36]. The findings also suggested that the convergent validity was stronger between the QMI and measures of marital satisfaction compared to the relationship with measures of anxiety, depression, and stress.

Consistent with previous studies [23, 25], infertility duration was significantly related to QMI scores. In addition, similar findings have been reported in other studies on measures of quality of life [37], anxiety, and depression [9, 23, 38]. Other demographic variables were not statistically related to QMI scores.

In summary, the QMI is a reliable and valid tool for measuring overall marital satisfaction in infertile patients. This inventory is a short and easy to use tool and can be administered in several minutes providing an economic tool for both research and clinical applications.

## Limitations

There are several limitations of the study that should be noted. First, the present study was a single-center research, thus, the generalization of the results may be limited. Second, the cross-sectional design limits our ability to make causal inferences between QMI scores and demographic and infertility characteristics. Third, the test–retest reliability of the QMI was not done among respondents.

## Data Availability

The datasets used and/or analyzed during the current study available from the corresponding author on reasonable request.

## Abbreviations

QMI: Quality of Marriage Index
KMSS: Kansas Marital Satisfaction Scale
RAS: Relationship Assessment Scale
CSI-4: Couples Satisfaction Index-4 Item
HADS: Hospital Anxiety and Depression Scale
PSS-4: Perceived Stress Scale-4 Item
CFA: confirmatory factor analysis
GFI: goodness of fit index
CFI: compara-tive fit index
NFI: normed fit index
RMSEA: root mean square error of approximation
SRMR: standardized root mean square residual
